# Tear biomarkers for keratoconus

**DOI:** 10.1186/s40662-016-0051-9

**Published:** 2016-08-04

**Authors:** Krishnatej Nishtala, Natasha Pahuja, Rohit Shetty, Rudy M. M. A. Nuijts, Arkasubhra Ghosh

**Affiliations:** 1 Cornea Department, Narayana Nethralaya, Bangalore, India; 2 GROW Research Laboratory, Narayana Nethralaya Foundation, Bangalore, India; 3 Cornea Clinic, Department of Ophthalmology, Maastricht University Medical Center, Maastricht, Netherlands

**Keywords:** Keratoconus, Tears, Biomarkers, Proteins, Metabolites

## Abstract

Keratoconus is a progressive corneal thinning, ectatic condition, which affects vision. Recent advances in corneal topography measurements has helped advance proper diagnosis of this condition and increased research and clinical interests in the disease etiopathogenesis. Considerable progress has been achieved in understanding the progression of the disease and tear fluid has played a major role in the progress. This review discusses the importance of tear fluid as a source of biomarker for keratoconus and how advances in technology have helped map the complexity of tears and thereby molecular readouts of the disease. Expanding knowledge of the tear proteome, lipidome and metabolome opened up new avenues to study keratoconus and to identify probable prognostic or diagnostic biomarkers for the disease. A multidimensional approach of analyzing tear fluid of patients layering on proteomics, lipidomics and metabolomics is necessary in effectively decoding keratoconus and thereby identifying targets for its treatment.

## Background

Keratoconus is a progressive, asymmetric corneal ectasia. It is characterized by the thinning and protrusion of the cornea leading to irregular astigmatism and myopia thereby affecting the visual performance [1].

### Clinical characteristics and diagnosis

The disease is clinically diagnosed by corneal thinning and steepening along with the presence of iron oxide hemosiderin deposits in corneal mid periphery (Fleischer’s ring). Around 50 % of subjects exhibit Vogt’s striae, which are fine stress lines caused by stretching and corneal thinning [2]. Although clinical signs are helpful for the diagnosis, keratoconus is typically confirmed by corneal topography or tomography [3]. Although keratoconus is bilateral, in certain scenarios, the clinical and topographic signs are present in only one eye (unilateral keratoconus) wherein the other eye is then diagnosed as form fruste keratoconus [4, 5]. The onset of the disease occurs at puberty and progresses up to the third decade of life [6, 7]. Hence, a severity grading has been proposed for keratoconus to differentiate a normal eye from a keratoconic eye based on the clinical signs and corneal topography indices [8, 9]. Keratoconus has been reported to affect 1 in 500 to 1 in 2000 individuals and has a greater prevalence in Asians indicating the role of ethnicity [5, 10–16].

### Causative factors

Both genetic and environmental factors contribute to the disease pathology [17–19]. Although atopy, eye rubbing, ocular allergies, Down’s syndrome and tapetoretinal degeneration have been associated with keratoconus [13], the etiology and pathogenesis still remains unclear. Despite the association of various genetic loci in twin and familial studies, [19] the general consensus remains that the disease is polygenic and is dependent on ocular surface and tear molecular expression changes [20].

### Tear proteomics

Tear fluid has been an important source of information in understanding ocular physiology [21]. A large number of proteases and protease inhibitors have been identified in tears [22]. Zhou et al., have identified over 1500 proteins in the tear fluid majorly involved in carbohydrate catabolism, proteolysis, protein transport besides immune response and regulation of apoptosis [23]. Disease specific molecular signature from tear fluid analysis can help in understanding the etiology of the disease and to help in prognosis. In addition, tear fluid can serve as an optimal source of molecular targets for treating ocular disease conditions [24].

In this review, we attempt to discuss, in a concise manner, the molecular markers for keratoconus that have been recently discovered and the importance of tear film as a source of these biomarkers in understanding the etiopathology as well as in prognosis of the disease.

## Review

### Structure of the tear film

Tear film is essential in maintaining ocular homeostasis and monitoring ocular surface conditions [25]. The tear fluid secreted by the lacrimal glands is a complex matrix comprising of an inner mucin layer, middle aqueous layer and the outer lipid layer and is composed of lipids, proteins, peptides, proteases, protease inhibitors and metabolites. The lipid layer provides lubrication, stability to the tear film and plays a protective role by preventing the cornea from drying and shields against pathogens [22, 26, 27]. Tear fluid helps in nourishing the corneal epithelium and anterior stroma by delivering nutrients and metabolic products [28]. Constitution of the tear film influenced by disease specific changes in the molecular markers can be of diagnostic value.

Another facet of the tear fluid analysis is the lipidome. Analysis of the tear fluid lipidome by Rantamaki et al. shows polar lipids, and the majority of tear lipids being phospholipids; the composition of tear lipids differs distinctly from those of the meibomian gland [29]. A more recent and comprehensive analysis of the human tear fluid identified more than 600 lipid species belonging to 17 major lipid classes [30]. Besides lipidomics, a metabolome analysis of tears from healthy subjects using mass spectrometry revealed 60 tear metabolites of different classes [31].

### Tear biomarker discovery

#### Tear fluid as a source of biomarkers

Proteins lipocalin (LCN), lysozyme (LYZ), lactoferrin (LTF), serum albumin (ALBU), proline rich repeat proteins (PRR) constitute the most abundant proteins in the tear fluid [32]. Inflammatory proteins were observed in tears of patients on long term glaucoma medication [33] and tear fluid proteins were also considered as a means for screening diabetic retinopathy [34, 35]. Tear proteins have been reported to be altered in patients with keratoconus with and without contact lens wear when compared to controls [36]. Tear composition has been shown to be altered in various inflammatory diseases like dry eye syndrome (DES) [37], keratoconus [38], primary open angle glaucoma [39] and Grave’s ophthalmopathy [21, 40]. Tear fluid has been studied as a source of biomarkers in the diagnosis of systemic conditions such as breast cancer [41, 42], diabetes [43, 44], multiple sclerosis [45], and severe acute respiratory syndrome (SARS) [46]. These reports highlight the importance of tears not only for the study of ocular conditions but also for readouts of systemic conditions.

Similar to the tear proteome, the tear lipidome and metabolome can also undergo changes in keratoconus as observed in other ocular disease such as DES where reduced levels of wax esters and saturated fatty acyl moieties in 93 DES patients were observed [47]. Besides lipids, the tear metabolome has also been studied to understand disease pathology. Using nuclear magnetic resonance (NMR) spectroscopy, levels of about 50 metabolites were found to be varying in the tears of 55 DES patients compared with healthy subjects [48]. Advances in technologies such as mass spectrometry and NMR have helped in studying and understanding molecular changes in the tear proteome, lipidome and metabolome relating to an ocular disease condition.

#### Maintenance and handling of tear fluid for biomarker discovery

Storage and maintenance of the collected tear fluid under proper temperature conditions is critical for successfully discovering a biomarker. Tear fluid from patients can be non-invasively collected either using a glass capillary or Schirmer’s strip [49]. However, the Schirmer’s strip method of collecting tears is considered the most effective with minimum discomfort to the patient and is often part of the DES ocular surface disease test that patients already undergo in the clinic. Since tear composition is complex with various proteolytic enzymes, cytokines, and metabolites, the tear fluid should be stored at sub-zero temperatures i.e., −70 to −80 °C. A reduction in total tear protein concentration was observed when stored at room temperature for 4–8 h [50].

### Biomarkers of keratoconus

#### Genetic risk factors/markers of keratoconus

Genetic variation is one of the factors influencing the incidence of keratoconus [19]. Genetic predisposition to keratoconus in familial and monozygotic twins has been well established [51, 52]. Single nucleotide polymorphisms (SNPs) in hepatocyte growth factor RAB 3 GTPase activating protein 1 (RAB3GAP1), interleukin 1β (IL1B), cadherin 11 (CDH11), negative regulator of ubiquitin like protein 1 (NUB1), collagen type XXVII A1 (COL27A1) and lysyl oxidase (LOX) were identified by genome wide association studies (GWAS) as risk factors for keratoconus [53]. However, the molecular functions associated with these SNPs are not well understood. Gene expression signatures of keratoconus have been reported and offer new insights into the disease process [20].

#### Gene and protein expression markers in cultured corneal cells and patient corneas

Differential gene expression analysis in debrided corneal epithelia of patients with keratoconus showed dysregulations of LOX and collagens - collagen I alpha 1 (COLIA1) and collagen IV alpha 1 (COLIVA1) and an elevated expression of matrix metalloproteinase 9 (MMP9) [54]. A positive correlation between the reduced collagen expression with clinical severity of keratoconus was observed indicating their role in structural deformity in keratoconic corneas [54]. These genes were also associated with elevated inflammatory cytokine IL-6 in the corneal epithelium and in the tears of keratoconus patients. However, cyclosporine A (CyA) treatment of tumor necrosis factor α (TNFα) stimulated corneal epithelial cells and inhibited the expression of IL-6, TNFα and MMP9 [55]. Cultured human keratocyte cells (HKCs) stimulated with three different isoforms of transforming growth factor β (TGFβ I-III) showed deregulation of SMAD6 and SMAD7, indicating altered TGFβ signaling in the progression of keratoconus [56]. Similarly a significant reduction in levels of alcohol dehydrogenase (class 1) betapolypeptide (ADH1B) in cultured keratoconic corneal fibroblasts suggested its possible role in keratoconus [57].

Reduced levels of beta actin, a protein essential for cell survival and growth, along with human antigen R (huR) was observed in keratoconic and normal corneas both at the transcriptional and translational level suggesting the deregulation of these molecules as a possible trigger for keratoconus [58]. Similar reduction in gene expression of beta actin and alpha enolase was also observed in superficial keratoconus epithelial cells compared with normal corneas, suggesting the degradation of these proteins in keratoconus [59]. Nielsen et al. performed a proteomic analysis using 2D-gel electrophoresis followed by mass spectrometry from keratoconus patient epithelia. Compared to controls, the patient epithelium overexpressed gelsolin (GSN), alpha enolase (ENOA), S100A4 and cytokeratin3 (KRT3), which suggests a plausible role of these proteins in the pathogenesis of keratoconus [60] while possibly associating the role of structural proteins and enzymes in keratoconus. These molecules can act as prognostic or diagnostic biomarkers and may aid in treatment of the disease. A list of the molecular markers identified in cultured corneal cells and human corneas are summarized in Table 1.

**Table 1:** Summarized list of biomarkers in keratoconus

Biomarker	Expression	Tissue/Cells	Technique/Method	Ref
LOX	↓	Patient Corneal epithelium	RT-PCR	47
COLIA1	↓			
COLIVA1	↓			
MMP9	↑	Patient Corneal epithelium	RT-PCR	48
TNFα	↑			
IL-6	↑			
SMAD6	↓	HKCs	RT-PCR/WB	50
SMAD7	↓			
ADH1B	↓	Cornea	Microarray/WB	51
GSN	↑	Patient corneal epithelium	2D-GE	53
ENOA	↑			
S100A4	↑			
KRT3	↑			

The genomic studies thus far have remained inconclusive in terms of a molecular explanation for the phenotypes observed and do not strongly correlate with the molecular expression data from patient corneas. However, the gene and protein expression data from the keratoconic cornea do show deregulation of important factors and pathways such as collagen synthesis, inflammation, extracellular matrix, structural genes, TGFβ pathway and Wnt signaling, to name a few. However, in a clinic, it is difficult to obtain corneal tissue for diagnosis and molecular profiling. This makes profiling tears of patients an attractive prospect.

### Tear specific markers for keratoconus

#### Pre-selected/targeted protein markers (ELISA)

Keratoconus is associated with tissue degradation that involves extracellular matrix remodeling, collagen deficiency [54] and more recently, increased roles of pro-inflammatory cytokines, cell adhesion molecules and matrix metalloproteinases [61]. Enzyme linked immunosorbent assay (ELISA) analysis of capillary collected tears in 28 [61], 30 [62] and 94 [63] patients with keratoconus in three different studies showed elevated levels of inflammatory markers IL-6, TNFα and MMP9. Thus, a variety of matrix metalloproteinases (MMP) -1, -3, -7, -13, interleukins (IL) -4, -5, -6, -8, and tumor necrosis factor (TNF) -α and -β are elevated in keratoconus tears [64]. Studies also demonstrate higher gelatinolytic and collagenolytic activities in keratoconus [65]. Hence, patients with elevated MMP9 levels, when treated with 0.05 % CyA for 6 months, showed improved corneal topography characterized by reduced disease progression, localized flattening and low tear MMP9 [55]. In another study where tears from 33 keratoconus patients were analyzed by ELISA, secreted-frizzled related protein 1 (SFRP1), a protein associated with the Wnt signaling pathway, was observed at lower levels compared to age-matched controls, indicating a novel signaling pathway alteration in keratoconus [66] (Table 2). Tear cytokine analysis performed to determine the effect of collagen cross-linking on tear fluid composition showed alterations in the tear cytokine levels [67]. These discoveries have significant clinical importance in aiding the development of biomarker kits targeted towards the diagnosis, prognosis and treatment of keratoconus as in the case of other ocular diseases such as DES. A tear MMP9 point-of-care detection kit was developed for the diagnosis and management of pre-op and post-op inflammation in DES [68]. Furthermore, pre-existing information based on different experimental methodologies enhances the reliability of pre-selected markers. The high specificity and sensitivity of target specific biochemical assays make them reliable and clinically relevant for detecting molecular changes both in a qualitative and quantitative manner.

**Table 2:** Summarized list of tear biomarkers in keratoconus

Protein	Expression	Ref
By ELISA Analysis		
IL-6	↑	48,55,56
TNFα	↑	
MMP9	↑	
MMPs-1,3,7,13	↑	57
IL-4,5,6,8	↑	
TNFα, β	↑	
SFRP1	↓	58
By LCMS Analysis		
SCGB2A1	↑	60
MMP1	↑	
AZGP1	↓	61
LTF	↓	
Cystatins	↓	62
LCN	↑	
AZGP1	↑	64
PIP	↑	

#### Mass spectrometry based protein markers

Mass spectrometric analysis for protein biomarkers has a dual advantage of surveying the unknown species thereby discovering novel molecular changes from a repertoire of proteins, and quantifying the changes in a multiplexed manner [69]. Using a nano-LC tandem mass spectrometry approach, tears of 44 keratoconus patients were compared with 20 healthy controls that identified the elevation of cytokeratins, matrix metalloproteinase 1 (MMP1) and mammoglobin B (SGB2A1) [70]. Additionally, lipocalin (LCN), lysozyme C (LYZ), immunoglobulin alpha & kappa (IGKA & IGKC) and precursors to prolactin were deregulated in keratoconus [70]. Tear analysis using a 2-DE/MS approach, identified zinc-α2-glycoprotein (AZGP1), immunoglobulin kappa chain, and lactoferrin (LTF) to be reduced in keratoconus [71]. Using complimentary proteomic approaches of 2DE and LCMS^E^ (mass spectral data acquired in a data- independent acquisition mode, MS^E^), Acera et al. have shown a significant decrease in the levels of cystatins and a higher tear lipocalin-1 in patients with keratoconus [38]. Moreover, Balasubramanium et al. demonstrated reduction in total tear protein in keratoconus patients [65]. Protein levels of gross cystic disease fluid protein-15/prolactin-inducible protein (PIP) and zinc-alpha-2-glycoprotein have been found to be elevated in tears of 36 patients by proteomic analysis, suggesting their application as prognostic markers for keratoconus [72] (Table 2).

One of the major challenges in tear proteomics is the wide dynamic range of tear proteins. As discussed earlier, certain proteins [32] due to their high abundance, are able to mask the identification of low abundant proteins. A comparison of the levels of abundant proteins in tears and serum showed tear fluid to be similar in its protein abundance to serum [23]. Though fractionation by 2DE helps in resolving the abundance and complexity, it is limited by other factors such as protein isoelectric point and hydrophobicity [73]. A gel-free approach overcomes such limitations and coupled with fractionation methods such as cation exchange chromatography, it aids in resolving sample abundance and complexity [23].

Metabolic changes as a consequence of disease can also be diagnosed from tears. Tear metabolome analysis by LCMS among 45 subjects in 3 clinically defined groups (healthy, keratoconus patients with RGP lens and keratoconus with no correction) identified 296 different metabolites among which more than 40 metabolites associated with glycolysis, gluconeogenesis and the urea cycle showed significant changes in keratoconus tears [74]. Thus, the changes in underlying molecular signaling and secretion pathways due to keratoconus could be disease specific, affecting numerous biological processes. Metabolite analysis by targeted mass spectrometry performed on 2D and 3D cultured human corneal keratocytes (KCKs), human corneal fibroblasts (HCFs) and HKCs detected about 150 metabolites, the majority of which are involved in the oxidative stress pathway, implying its importance in keratoconus [75].

#### Disease specificity of tear biomarkers

Tear proteins LYZ, LTF, LCN were also reported to be downregulated in DES. Besides, tear cytokines IL -4, -5 and -6, and TNFα, which were elevated in keratoconus, were also shown to have similar expressions in DES [76, 77], indicating certain similar molecular changes among the two disease conditions. Hence, it is imperative to practice caution while considering tear molecular changes as biomarkers for a specific disease. Albeit, recent studies have shown that matrix metalloproteinases MMP1 and 9, AZGP1, SFRP1, IGKC and cell adhesion molecules ICAM-1 and VCAM-1 to be specifically regulated in keratoconus [36, 71], indicating their probable exclusive role in keratoconus. In addition, the reduced activity of LOX [54] and levels of SFRP1 [66] are specific to the disease and highlight the molecular pathways that can be targeted for disease correction. Since collagen cross-linking is the current surgical treatment available for keratoconus [78], reduction in the natural collagen cross linkers [54] is perhaps expected. The molecular pathways leading to the elevation of collagenolytic factors [55] presents another attractive target for therapy. Since these large sample studies [54, 63] indicate a broad range in the expression of such factors, it is imperative to tune future treatment modalities based on specific molecular targets to the correct pathways. This could be possible through tear-based biomarker profiling.

It is now understood that inflammation is one of the primary drivers of keratoconus [55, 61–63, 71], therefore, it is not surprising that some of the markers between keratoconus and DES are similar. However, the amplitude of the deregulation of these markers such as IL6 and MMP9, in correlation with disease phenotype can be distinct amongst the different disorders with inflammatory drivers. Large sample cohort studies [54, 63] would further validate the role of these proteins as specific and potential biomarkers for keratoconus. Therefore, accumulated information about these markers should be considered for application in diagnosis e.g., elevated MMP9 and IL6 [55] along with a reduction in markers such as LOX, SFRP1 [54, 66] in tears can give a specific diagnosis of keratoconus. Many of these factors are deregulated at the corneal tissue (epithelium and stroma) level as well as the tears, further validating their importance as biological processes specific to this disease as established by different groups.

With the advent of high throughput technologies such as multiplex cytokine bead array [77] and quantitative mass spectrometry by multiple reaction monitoring (MRM) assays, multiple candidate markers can be validated in a relatively shorter time and over a larger cohort of patients. Zhou et al. have demonstrated quantitation of 47 tear proteins in a MRM assay [69]. Multiplexed validation also establishes a unique molecular fingerprint of the disease and a more appropriate way of diagnosing and treating keratoconus.

## Conclusions

Tear fluid as a source of biomarkers in ocular and systemic conditions has been well studied and is shown to have translational potential. Moreover, the non-invasive way of collecting the tears makes it an optimal biological fluid to study with minimal to no discomfort to the patient. Studies performed on tear fluid in patients of keratoconus provided insights into the pathology of the disease and has revealed probable prognostic as well as diagnostic biomarkers for the disease. More importantly, the recent studies and data from tear analysis establishes the definitive role of inflammation as a driver of corneal collagen loss and deformity in keratoconus patients. Elevated cytokines move in tandem with elevated matrix degrading enzymes to reduce levels of collagens and collagen crosslinking enzymes resulting in the disease. We term this as “Keratoconus Inflammaxis”, a unique molecular signaling fingerprint identified with this disease and its related pathology. The advances in technology such as high resolution high sensitivity mass spectrometry have enabled mapping of the tear fluid in patients and also the heterogeneity of keratoconus pathology. A multi-omics approach integrating data from proteomics, lipidomics and metabolomics is the need of the hour for studying tear fluid as an important source of biomarkers in keratoconus to lead to effective prognosis and treatment of the disease.
